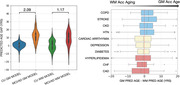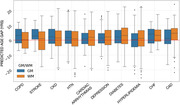# Chronic Medical Conditions and Dementia Risk: Brain Age Models for Quantifying Impact and Understanding Mechanisms

**DOI:** 10.1002/alz.093829

**Published:** 2025-01-09

**Authors:** Xi Yu, Scott A. Przybelski, Robert I. Reid, Robel K Gebre, Timothy G. Lesnick, Maria Vassilaki, Jonathan Graff‐Radford, David S. Knopman, Ronald C. Petersen, Clifford R. Jack, Prashanthi Vemuri

**Affiliations:** ^1^ Mayo Clinic, Rochester, MN USA; ^2^ Department of Quantitative Health Sciences, Mayo Clinic, Rochester, MN USA; ^3^ Mayo Clinic, Quantitative Health Sciences, Rochester, MN USA; ^4^ Department of Neurology, Mayo Clinic, Rochester, MN USA; ^5^ Department of Radiology, Mayo Clinic, Rochester, MN USA

## Abstract

**Background:**

Chronic medical conditions increase dementia risk, and the mechanism may be captured computationally by measures of accelerated brain aging. We hypothesized that quantifying the impact of chronic conditions on GM and WM aging will aid in measuring the differential impact of each condition on brain aging and shed light on the mechanisms through which they increase dementia risk. We specifically focused on microstructural brain changes with Neurite Orientation Dispersion and Density Imaging (NODDI) based on advanced diffusion MRI ‐ neurite density index (NDI), free water fraction (FWF), and orientation dispersion index (ODI).

**Method:**

We identified 1123 participants [913 cognitively unimpaired (CU), 114 with MCI, 96 with AD] from the Mayo Clinic Study of Aging (MCSA) and Mayo ADRC with multi‐shell diffusion MRI. MCSA participants had chronic medical conditions’ diagnosis (based on US Dept of HHS) extracted from the electronic health care records. Using the BorutaSHAP algorithm on NDI, ODI, and ISOVF features from GM and WM separately, we constructed two distinct brain age models for GM and WM. First, we evaluated the usefulness of the predicted age gap (PAG), which is the difference between predicted age by model and chronological age, for both WM and GM NODDI models in CU/MCI/AD. We then developed separate GM and WM models for each condition using samples from condition‐negative participants to compare PAG of GM and WM for that condition.

**Result:**

There was greater PAG in GM (avg = ) than WM (avg = 9.15 yrs) in MCI/AD participants (Fig. 1A). Each condition shifted the GM PAG by 0.10 to 3.56 years and WM PAG by 0.38 to 3.72 years with stroke and congestive heart failure showing the greater PAG overall for GM and WM PAD respectively (Fig. 2). COPD, stroke, chronic kidney disease, hypertension had comparatively greater GM PAG than WM PAG; while hyperlipidemia, congestive heart failure and coronary artery disease had greater WM PAG than GM PAG (Fig. 1B).

**Conclusion:**

Chronic conditions are associated with increased GM and WM Brain aging models can aid in quantifying the impact of chronic conditions on dementia risk and understanding mechanisms.